# Spatial Analysis of Socio-Economic and Demographic Factors Associated with Contraceptive Use among Women of Childbearing Age in Rwanda

**DOI:** 10.3390/ijerph15112383

**Published:** 2018-10-27

**Authors:** Faustin Habyarimana, Shaun Ramroop

**Affiliations:** 1 School of Mathematics, Statistics and Computer Sciences, University of KwaZulu-Natal, Pietermaritzburg Campus, Private Bag X01, Scottsville 3209, South Africa; ramroops@ukzn.ac.za; 2 College of Education, University of Rwanda, PO BOX 5039 Kigali, Rwanda

**Keywords:** family planning, Bayesian, spatial, RDHS, Rwanda

## Abstract

Contraceptive use is considered as essential for protecting women’s health and rights, influencing fertility and population growth, and helping to promote economic development. The main objective of this study was to analysis the factors and spatial correlates of contraceptive use among women of childbearing age. The 2015 Rwanda Demographic and Health Survey (RDHS) data were used to identify the factors associated with contraceptive use in Rwanda. A Bayesian geo-additive model was used in order to account for fixed effects, nonlinear effects, spatial and random effects inherent in the data. The overall prevalence of use of any contraceptive method among married women of childbearing age in Rwanda was 52.7%. A woman’s age, wealth quintile, level of education, working status, number of living children, and exposure to the media was found to increase contraceptive use. The findings from the study also found disparities in contraceptive use at provincial and district level, where prevalence was higher in districts of Northern provinces and lower in districts of western provinces. The findings of this study suggest that exposure to information on contraceptive use in health centres, empowerment of women to access quality contraceptive-use services and religions to play an important role in explaining and informing their adherents on the importance of using a contraceptive method.

## 1. Introduction

Contraceptive use is considered as essential for protecting women’s health and rights, influencing fertility and population growth, and helping to promote economic development, especially in sub-Saharan Africa. Contraceptive use helps to avoid maternal deaths by preventing unwanted pregnancies and abortions. It also helps to determine the number of children in the family and enhances adequate child spacing [1,2]. Some contraceptive methods such as condoms help, not only for family planning purposes, but also to prevent sexually transmitted infections, for instance, HIV, among others.

The various studies in the literature point out that satisfying a woman’s need for contraception, particularly in family planning, may considerably reduce fertility [3,4]. However, young women confront many problems in contraceptive method use, mainly in family planning services, for instance, fear of side effects, cost, and lack of enough information [5]. In general, contraception use and family planning are essential for improving the health of the population. Although many United Nations member countries, especially those in the developed world, have strong family planning programmes, this is not the case in some sub-Saharan African countries, some sub-regions of Asia and Latin America and in the Caribbean, where, regardless of a rise in the contraceptive-prevalence rate, a large number of mainly poor, uneducated women and those with limited access to family planning services, continue to have unmet needs for contraceptives [6,7].

The prevalence of any type of contraception-use method among married women in Rwanda has improved in the last fifteen years. It was 17.4% among married women in 2005, tripled (52%) between 2005 and 2010, and was 53% in 2014 [8]. This may be attributable to the Government of Rwanda’s commitment to strengthen the health sector, especially family planning services. The maternal and child mortality rate in Rwanda has also tremendously declined over a period of 20 years. As shown by the Rwanda Demographic and Health Survey reports, in 2000, 2005, 2010 and 2015 there were 1 071, 750, 476 and 210 deaths per 100,000 live births respectively for maternal and for child mortality [8]. In neighboring countries, especially in East Africa, in the last 15 years contraceptive use has generally increased. The prevalence of current contraceptive use among married women in Uganda was 26% in 2004, 34% in 2010 and 38% in 2016; in Tanzania it was 26.4% in 2005, 34.4% in 2010 and 38.4% in 2015 and 2016; in Kenya, it was 39% in 2003, 46% in 2008 and 2009 and 58% in 2014; and in Burundi it was 27.7% in 2010 and 29% in 2016 and 2017 [9,10,11,12]. Identifying determinants of contraceptive use among women of reproductive age is essential in order to formulate adequate health programmes, policies and strategies and possible interventions that can promote the well-being of children and maternal health in general. We now consider the problem of contraceptive use.

Various studies in the literature assessed the determinants of modern contraceptive (pill, IUD, injections, condoms, female sterilization, male sterilization, implants/norplant, lactational amenorrhea, standards days methods) use only [13,14,15] and did not include traditional methods (withdrawal, periodic abstinence) which may sometimes be the most accessible. The current study addresses this problem by combining all types of contraceptive-use methods among married women of childbearing age, nationwide. In addition, the RDHS data set has inherent nonlinear, spatial and random effects that needed to be accounted for. We consider these aspects to capture the heterogeneity of the data. Consequently, the main objectives of the current study were to address these problems in their entirety, by analyzing the factors associated with any type of contraceptive use and mapping the spatial distribution of use at district level in Rwanda. These objectives were achieved by applying structured spatial modelling that accounted for fixed, nonlinear, spatial and random effects. To the best of our knowledge, there is no study in the literature that assessed the determinants of contraceptive use among women in Rwanda, using a structured spatial model. It was expected that the findings from the present study would help policy makers and other public health institutions in Rwanda to visualize the spatial distribution of the use of any type of contraceptive method among women of reproductive age at district level. Therefore, it would help them to improve the current strategies when targeting the districts of low prevalence of contraceptive use.

## 2. Materials and Methods

### 2.1. Source of Data

The current study used data from the 2014/15 Rwanda Demographic and Health Survey (RDHS). The sampling used in this survey was a two-stage stratified method; in the first stage, 492 primary sampling areas were selected, whose 113 were selected from urban areas and 379 from rural areas. The sampling was done with probability proportional to the number of households in the village. In the second stage, systematic sampling was used for all households existing in the selected village and 26 households were selected in each village. More details on sampling techniques and data collection can be found in [8].

#### 2.1.1. Outcome Variable

The different types of contraceptive methods used are the modern (the pill, IUD, injection, male condom, female condom, female sterilization, male sterilization, implants/Norplant, lactational amenorrhea (LAM), the standard days method, periodic abstinence), traditional (withdrawal and periodic abstinence). In the survey, women were asked whether they used any of these methods. In this study, a woman is considered as a current contraceptive user if she uses at least one of the above-mentioned methods and as a non-contraceptive user if otherwise, and this was coded as “1” and “0” for contraceptive use and non-contraceptive use respectively.

#### 2.1.2. Independent Variables

The independent variables used in the current study have been used elsewhere [6,16,17,18,19]. The various socio-economic and demographic factors considered include current age of the respondent (continuous); the age at first cohabitation (continuous), level of education (no education, primary, secondary, tertiary); have heard about family planning in the last 12 months from newspapers/magazines (yes or no); have heard about family planning in the last 12 months on television (yes or no); have been visited by a family planning worker during the last 12 months (yes or no); husband or partner desire for more children compared to the wife or partner (same, husband wants more, husband wants fewer, do not know); wealth quintile of her household (poorest, poorer, middle, richer, richest); currently working (yes or no); province of residence (Kigali, South, West, North, East); place of residence (urban, rural); religious affiliation (Catholic, Protestant, Seventh Day Adventist, other); husband or partner’s education level (no education, primary, secondary, tertiary); decision-making for using contraceptive (mainly respondent, mainly husband or partner, joint decision, other); person who usually decides on healthcare (respondent alone, respondent and husband/partner, respondent and other person, husband/partner alone, someone else, other); number of living children (0, 1, 2, 3, 4 and more); and age of the husband or partner.

### 2.2. Statistical Model

Let $y_{ijm}$ denote the contraceptive use status of woman, *i* from stratum *j* and cluster *m*, with i=1, 2, 3, …, 6847, j=1, 2, 3, …, 60 and m=1, 2, 3, …, 492. The outcome variable is defined as a dichotomous variable such that $y_{ijm}=1$ if the women *i* is currently using any type of contraceptive method and $y_{ijm}=0$ if the women *i* is not currently using any type of contraceptive method. The contraceptive use status among women of reproductive age is a binary outcome in the current study and hence it is assumed to follow a Bernoulli distribution:

(1) $$y_{ijm}\sim Bernoulli\left(p_{ij}\right)$$

where $p_{ij}$ is the probability that a woman i from district j is currently using a contraceptive method and $1-p_{ij}$ is the probability that a woman i from district j is not currently using a contraceptive method. Therefore, the use of contraceptive methods among women of reproductive age can be associated with the explanatory variables using an appropriate link function from a generalized linear models approach as follows:

(2) $$logit\;\left(p_{ij}\right)=\log\left(\frac{p_{ij}}{1-p_{ij}}\right)=W_{ij}^{\prime }\beta$$

Model (2) is known as binary logistic regression, where $W_{ij}^{\prime }$ is the vector of explanatory variables and β is the vector of coefficient parameters. However, classical Generalized Linear model (GLM) has a rigid assumption that all observations are independent; but this assumption is sometimes not satisfied, as some observations may have, for instance, spatial dependence, or may have nonlinear effects. Hence, there is a need to include nonlinear and spatial variability in model (2) and it is given by:

(3) $$logit\;\left(p_{ij}\right)=W_{ij}^{\prime }\beta +\overset{q}{\underset{k=1}{\sum }}f_{k}\left(x_{ijk}\right)+f_{spat}\left(s_{j}\right)$$

where $\beta_{i}$ is the vector of fixed effect corresponding to categorical variables, $f_{k}$ is the appropriate smooth function of continuous variables such as mother’s current age and mother’s age at first cohabitation and $f_{spat}\left(s_{j}\right)$ are the parameters of random effects, which capture unobserved spatial heterogeneity at district $s_{j}$.

#### 2.2.1. Parameter Estimation

In the current study, the parameter estimation is done based on a full Bayesian analysis framework, where the appropriate prior distributions have to be assigned to all unknown parameters. In this study, diffuse priors p(β)∝const are assigned to all fixed regression parameters and the second-order Gaussian random walk priors were assigned to non-parametric continuous covariates [20]. The structured spatial effects $s_{i}$ were modeled through a Gaussian Markov random field specified as an intrinsic conditional autoregressive (ICAR) prior distribution [21].

#### 2.2.2. Posterior and Inferences

Posterior distributions are the distribution of the parameters after observing the data and are obtained by updating the prior distribution with observed data. A full Bayesian inference is based on the analysis of the posterior distribution of the model parameters. In the current study, the Bayesian posterior marginal distribution was estimated, based on integrated nested Laplace Approximation [22] and R-INLA was used for inferential analysis.

#### 2.2.3. Model Selection

The model goodness of fit was assessed based on the Deviance Information Criteria (DIC) that states that the smaller the value of the DIC the better is the model fit [23,24] and the DIC value is given by:

(4) $$\mathrm{DIC}=\bar{D}+\mathrm{pD}$$

where $\bar{D}$ is the posterior mean of the deviance and pD is the number of effective parameters in the model that penalize the complexity of the model. The DIC takes both the model fit (summarized by $\bar{D}$) and the model complexity (captured by pD) into consideration when comparing models.

#### 2.2.4. Model Building

The present study fitted and examined the following models:

Model 1: $logit\;\left(p_{ij}\right)=W_{ij}^{\prime }\beta$

Model 2: $logit\left(p_{ij}\right)$ = $W_{ij}^{\prime }\beta +f_{1}\left(current\;age_{i}\right)+f_{2}\left(age\;at\;first\;cohabitation_{i}\right)$

Model 3: $logit\left(p_{ij}\right)=W_{ij}^{\prime }\beta +f_{spatj}\left(district_{j}\right),\;j=1,2,\ldots ,30$

Model 4: $logit\;\left(p_{ij}\right)=W_{ij}^{\prime }\beta +f_{1}\left(current\;age_{i}\right)+f_{2}\left(age\;at\;first\;cohabitation_{i}\right)+f_{j}\left(district_{j}\right),\;j=1,2,\ldots ,30$

Model 1 is classical logistic regression, where all categorical variables (women’s education, women’s region affiliation, women’s working status, women’s province of residence, have heard about family planning on radio in last 12 months, have heard about family planning in newspapers/magazines in last 12 months, visited by a family planning worker in the last 12 months, visited a health facility in the last 12 months, currently residing with husband or not, number of living children, wealth quintile of the household, husband desires children) and (current age of the woman and woman’s age at first cohabitation) were considered as fixed effects and assumed to have a linear effect on the outcome variable.

In model 2, categorical variables listed earlier in model 1 were assumed to have a linear effect on the response variable, whereas continuous variables were modeled non-parametrically and model 2 is commonly known as an additive logistic regression model. In model 3, all predictor variables were modeled as fixed effects and structured random effects as structured spatial effects that cover the unobserved covariates which are essential within the districts. Model 4 is an extension of model 2, including structured spatial effects and is known as a structured additive regression model.

## 3. Results

### 3.1. Descriptive Analysis

Table 1 presents descriptive statistics of the participants. The overall prevalence of any contraceptive use among married women of reproductive age in Rwanda was 52.7%, of whom 46.8% use modern methods and 5.9% use traditional methods. The average current age of women was 32.8 years, with a minimum age of 16 and a maximum age of 49 years. The average age of first cohabitation was 21 years, the minimum age was 10 and maximum age was 95. Most of the respondents were from rural areas (83%) and 17.0% were from urban areas. It is also observed in the same table that 55.0% of respondents had heard about family planning on the radio, 7.1% on TV and 6.1% in newspapers or magazines. Table 1 shows that 29.2% of the respondents were visited by a family planning planner in the 12 months prior to the survey, 71.8% of the respondents visited health facilities in the 12 months prior to the survey and 46.3% were told about family planning at a health facility. The majority of the respondents (86.8%) were working during the period of the survey. It can also be observed that most of the respondents had primary education (70.5%) and most of the women had two living children (45.2%). It is also observed that 38.2% of the respondents were Catholic, 45.2% were Protestant, 3.2% were from religions other than Catholic, Protestant or Seventh Day Adventist and 12.8% were Seventh Day Adventist.

It is observed from Table 2 that modern contraceptive methods were the most (46.9%) used among married women of childbearing age in Rwanda and traditional method use was 5.9%.

The association between current contraceptive use among married women or women living with their partners, and various potential factors, was tested by the Chi-square statistical test at 5% level of significance and the results are summarized in Table 3. Education level of the respondents was significantly associated with current use of contraceptive methods (*p*-value < 0.0001). It can be seen from the table that the prevalence of contraceptive use was 56.8%, 53.5%, 53.7% and 47.3% among women with tertiary education, secondary and primary education level respectively. The working status of the woman was significantly associated with current use of contraceptive methods among women of reproductive age (*p*-value < 0.0001). It can be observed from the table that the prevalence of contraceptive use was 53.6% among women who were working at the time of the survey. Hearing about family planning on the radio, from newspapers or magazines and on the TV was each significantly associated with contraceptive use (*p*-value < 0.0001) and the prevalence of contraceptive use in this regard was 55.8%, 63.9% or 60.1% among the women respectively. Religion was significantly associated with contraceptive use among women. The prevalence of contraceptive use was 56.9% among Catholic women, 47.5% among Protestant women, 58.9% among Seventh Day Adventist women and 53.4% among women from religions other than Catholic, Protestant and Seventh Day Adventist. The issue of education and its influence on contraceptive use is also a salient finding of the current research. The education of the husband was significantly associated with current contraceptive use among women (*p*-value = 0.018). The prevalence of contraceptive use was 48.1%, 53.7%, 53.0% and 54.2% among women married to husbands with no education, primary education, secondary education and tertiary education respectively.

We now consider the model fit comparisons. The findings from Table 4 reveal that model 4 has smaller DIC compared to the DIC of model 1, model 2 and model 3. Therefore based on the principles of the DIC (that state that the smaller the DIC the better the model fit [23]), model 4 was found to be the better model fit, and it is therefore used in the final analysis in this study.

### 3.2. Fixed Effect

In the multivariate structured geo-additive model, any variable that was statistically significant in cross-tabulation at 5% level of significance were included in the analysis and the final analysis retained only those variables that were at 5% level of significance.

The results from fixed effects are summarized in Table 5. It is observed that the contraceptive use among women of reproductive age in Rwanda increases with higher wealth and education levels, exposure to mass media and number of living children. The education level of the woman is highly associated with contraceptive use among women of reproductive age. It was observed from the table that a woman with tertiary education was 79.3% (OR = 1.7934, 95% CI: 1.2355, 2.61300) more likely to use contraceptive methods compared to woman with no education. A woman with secondary education was 1.34 (OR = 1.3371, CI: 1.0853, 1.7331) more likely to use contraceptive methods compared to a woman without education, while a woman with primary education was 1.17 (OR = 1.1735, CI: 1.0125, 1.3599) more likely to use contraceptive methods compared to a woman without education.

The wealth of the family was found to be a significant predictor of women’s contraceptive use. Women from richest (OR = 1.4330, CI: 1.1727, 1.7517), richer (OR = 1.3496, CI: 1.1340, 1.6063) and middle (OR = 1.2462, CI: 1.0533, 1.4745) families were more likely to use contraceptive methods than women from poorest families.

Considering health facilities, a woman who visited a health facility within 12 months prior to the survey was 1.2165 (OR = 1.2165, 95% CI: 1.0882, 1.3681) more likely to use any type of contraceptive method compared with a woman who did not visit a health facility within 12 months.

It was also observed from the results that a woman who was not residing with a husband or partner at the time of the survey was 0.559 (OR = 0.5593, 95% CI: 0.4702, 0.6647) times less likely to use contraceptive methods compared to a woman who was residing with a husband or partner. It was also observed that contraceptive use increases with the number of living children in the family. A woman who had no living child was 0.0072 (OR = 0.0072, 95% CI: 0.0029, 0.0156) less likely to use a contraceptive method compared with a woman who had four or more living children. A woman who had one living child was 0.447 (OR = 0.4468, 95% CI: 0.3544, 0.5625) less likely to use a contraceptive method compared to a woman who had four or more living children. A woman who had two living children was 0.737 (OR = 0.7368, 95% CI: 0.6131, 0.8848) less likely to use a contraceptive method compared to a woman who had four or more living children. A Catholic woman was 0.7047 (OR = 0.7047, 95% CI: 0.6308, 0.7936) less likely to use a contraceptive method compared to women from other religions other than Protestant and Seventh Day Adventist. A working woman was 1.27 (OR = 1.2705, 95% CI: 1.0868, 1.4850) more likely to use a contraceptive method than a non-working woman. A woman who had heard about family planning on the radio was 1.1173 (OR = 1.1173, 95% CI: 1.0021, 1.2455), on TV was 1.2200 (OR = 1.2200, 95%CI: 1.0812, 1.3681) and from newspapers/magazines was 1.3343 (OR = 1.3343, 95%CI: 1.0564, 1.6896) more likely to use a contraceptive method than a woman who had not heard about family planning within 12 months. Contraceptive use is higher in the Northern Province compared to other provinces. A woman from Northern Province was 1.43 (OR = 1.4305, CI: 1.1436, 1.7898) more likely to use a contraceptive method than a woman from the Western Province, but a woman from the Southern Province, Kigali and East did not show any statistical association. A woman whose husband desired more children than her was 1.2998 (OR = 1.2998, 95% CI: 1.1069, 1.5258) more likely to use a contraceptive method than a woman whose husband desired the same number of children. A woman who did not know whether her husband or partner desired the same, less or more children was 1.230 (OR = 1.2304, 95% CI: 1.0182, 1.4866) more likely to use a contraceptive method than a woman whose husband desired the same number of children.

### 3.3. Non-Linear Effect

**Figure 1:**
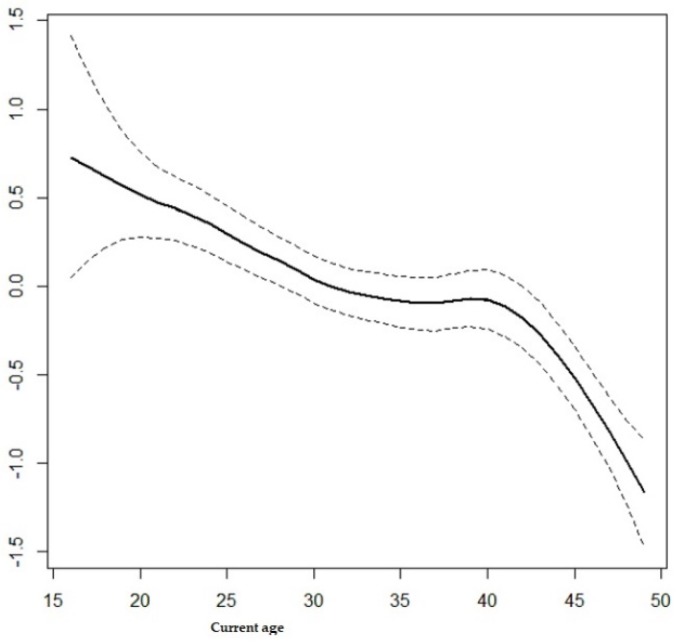
Effects of woman’s current age on the use of contraceptive methods.

**Figure 2:**
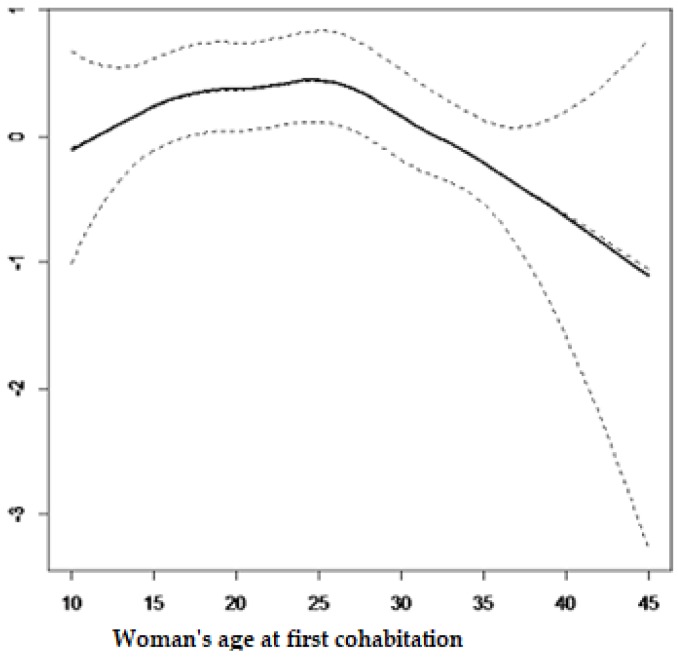
Effects of woman’s age at first cohabitation.

The current study also considered the non-linear effects from continuous variables on contraceptive use and results from the structured multivariate model are summarized in Figure 1 and Figure 2. It is observed from Figure 1 that contraceptive use among women of reproductive age decreases with age. It can be observed from Figure 2 that contraceptive use follows an inverse U-shape, where the contraceptive use increases with the woman’s age at first cohabitation, up until close to 25 years old and afterward decreasing with age.

### 3.4. Spatial Effects

**Figure 3:**
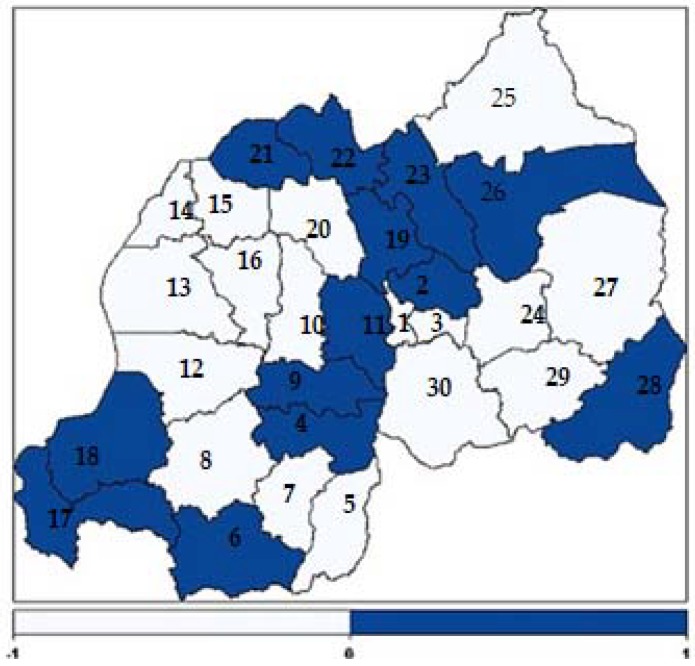
Structured spatial effects on contraceptive use among women of childbearing age.

The present study found positive and negative structured spatial effects on contraceptive use among women of reproductive age in Rwanda. Figure 3 presents structured spatial effects on contraceptive use among women of childbearing age. The light blue colour shows low contraceptive use while dark blue shows high contraceptive use. The numbers (1-30) shown in the map correspond to the districts codes indicated in Table 6.

## 4. Discussion

The main objective of this study was to identify the factors associated with contraceptive use among women of childbearing age in Rwanda and to identify and map the possible spatial distribution of contraceptive use at district level. Figure 3 shows that contraceptive use was unequally distributed among districts, being higher in districts of the Northern Province and lower in districts of the Western Province. This finding is similar to the findings in [8]. In the dark blue coloured district a great number of women use contraceptive methods and in the light blue coloured districts a low number of women use contraceptive methods. Figure 3 shows that contraceptive use among women in the dark blue coloured areas was higher, mostly in four districts of the Northern Province (Musanze, Burera, Rulindo, Gicumbi), in four districts from the Southern Province (Kamonyi, Ruhango, Nyanza and Nyaruguru), in two districts from the Eastern Province (Kirehe and Gatsibo), in two districts from Western Province (Nyamasheke and Rusizi) and in Gasabo district in Kigali City [8].

The level of wealth of the family was found to be a significant predictor of contraceptive use among women of reproductive age. The findings from this study indicated that contraceptive use increases with greater wealth of the household. This finding is similar to that of [8,25,26]. This may be due to the fact that the wealthier families can easily access health centre facilities, mass media and education, among other factors, all of which are well known for increasing the use of contraceptives among women.

Exposure to the use of contraceptives was found to play an essential role in its use as well as in family planning. Similar findings were also found in studies by [15,19,27,28]. This may be due to the fact that, for instance, when a woman visits a health centre or is visited by health centre workers or family planning field workers, she is told about the importance of using contraceptive methods. This may explain why a woman in such cases was more likely to use a contraceptive method.

The current findings show a strong association between the number of living children in the family and use of contraceptive methods. It was observed from the results that contraceptive use increases with increasing numbers of living children. This was also found by [16,29,30,31,32]. A woman who has many children tends to limit births because of financial burdens of paying school fees and providing health care, among other factors.

The findings from this study pointed towards a significant relationship between religious affiliation and the use of a contraceptive method among women of reproductive age. This was also found in the studies by [33]. In this study, the use of contraceptives was highest among Protestant women, followed by Seventh Day Adventists. Among Catholic women there was not a significant association with contraceptive use compared to women from religions other than Protestant and Seventh Day Adventist. However, in some studies such as [34,35], a significant relationship was found.

The findings from the current study also highlighted a strong association between the education level of the women and contraceptive use. The results showed that the use of any type of contraceptive method increases with women’s level of education i.e., educated women are more frequent users of contraceptive use than uneducated women. This result was found in other similar studies [26,27,36,37,38]. However, studies by [26] in Mali found no significant association between education level of the women and contraceptive use. The significant association is not surprising because education is known as a powerful factor associated with women’s empowerment, knowledge about their body and reproductive physiology and other maternal and child health information. The educated women are most likely to perceive the advantage of having few children and its effect on the family or their individual economic productivity.

The results from the current study also revealed a strong association between working status of the women and the use of contraceptive methods among those of childbearing age in Rwanda. The women that were working at the time of the survey were more likely to use contraceptives compared to women who were not working over this period. This was found elsewhere in similar studies [33]. This may be due to the fact that working status sometimes empowers women, not only financially, but also to access contraception information, maternal and child healthcare and this empowerment promotes contraceptive use, especially for family planning.

As was expected, this study revealed a strong, significant association between contraceptive use and marital status. It was found that women who resided with their husband/partner were more likely to use any type of contraceptive methods than women who did not reside with their husband or partner. This was found in other studies by [27].

In light of the findings of the current study, the use of any contraceptive method reduces with increasing age, up to 25 years old, and thereafter, increases up to 42 years old, but thereafter decreases once more. In many similar studies in the literature it was also found that the use of contraceptives reduces with the increasing age of the women [32,36,39]. The low contraceptive use among women from the less than 30-year-old age group may be due to the fact that they are usually newly married and interested in having children in the first years of their marriage. The higher prevalence rate of contraceptive use among women aged between 30 and 42 years old may indicate that most of the women in this age group have reached their desired number of children. It is not surprising that after the age of 42 years, the use of contraceptive methods decreases, likely because many women at this age may not be sexually active. This may be due to the fact that a decrease in fecundity correlates with the onset of menopause.

The findings from the current study revealed that the age of the women at first cohabitation was a very important factor associated with the use of contraceptives among women of childbearing age. This was found elsewhere by [39]. Figure 2 showed that cohabitating at an early age increases the use of contraceptive methods. It was found that contraceptive use increases in the first cohabitation age group of 10 to 25 years old. This may be due to the fact that at this age a higher number of women are not yet married and may not desire children.

## 5. Study Limitations

The analysis in this study was mainly based on a cross-sectional study and this may not draw the causal relationship or effect between contraceptive use and independent variables; only associations can be drawn from this study. Therefore, a longitudinal study is suggested for future work in order to identify relevant trends and patterns over time. Furthermore, the study used on married woman only and not on unmarried women. Another possible area for future study is to differentiate types of birth control, as not all have equal effectiveness.

## 6. Conclusions

The findings of this study suggest that improvement of exposure to contraceptive-use information in health centres, empowerment of women to access quality contraceptive-use services and religious affiliation all play a significant role in explaining and informing their adherents on the importance of using a contraceptive method. These findings also highlighted the districts with lower numbers of women using contraceptive methods and this can help policy makers and other related public health institutions to design specific programmes targeting these districts in order to improve the health status and living conditions of these women.

## Figures and Tables

**Table 1:** Descriptive statistics of the participants’ details.

Variable	Categories	% or M or Range
**Current contraceptive use**	Yes	52.7
	No	47.3
**Respondent current age**	Continuous	M = 32.81 (Minimum = 16, maximum = 49)
**Age at first cohabitation**	Continuous	M = 21.09 (Minimum = 10, Maximum = 95)
**Place of residence**	Urban	17
	Rural	83
**Respondent education level**	No education	16.5
	Primary	70.5
	Secondary	10.3
	Tertiary	2.7
**Wealth index**	Poorest	18.8
	Poorer	21.2
	Middle	20.9
	Rich	19.9
	Richest	19.3
**Heard about family planning on the radio in last few months**	Yes	55
	No	45
**Heard about family planning on the TV in last few months**	Yes	7.1
	No	92.8
**Heard about family planning from newspapers/magazines in the last few months**	Yes	6.1
	No	93.9
**Visited by family planning worker in the last 12 months**	Yes	29.2
	No	70.8
**Visited health facility in the last 12 months**	Yes	71.8
	No	28.2
**At health facility, told of family planning**	Yes	46.3
	No	53.7
**Respondent currently working**	Yes	86.8
	No	13.2
**Husband/partner education level**	No education	16.9
	Primary	70.1
	Secondary	9.2
	Tertiary	3.5
	Do not know	0.2
**Number of living children**	0	5.2
	1	17.9
	2	45.2
	3	17.4
	4 or more	37.4
**Religion**	Catholic	38.2
	Protestant	45.6
	Seventh Day Adventist	12.8
	Others	3.2

**Table 2:** Prevalence of contraceptive use by type.

Current use by Method Type	N (%)
No method	3237 (47.3)
Traditional method	402 (5.9)
Modern method	3208 (46.8)

**Table 3:** The prevalence of contraceptive use among women of childbearing age by category.

Indicator Variable	Category	Contraceptive Use		*p*-Value
		No	Yes	
Woman’s education level	No education	596(52.7%)	535(47.3%)	<0.001
	Primary	2233(46.3%)	2592(53.7%)	
	Secondary	329(46.5%)	379(53.5%)	
	Tertiary	79(43.2%)	104(56.8%)	
Respondent currently working	Yes	2755(46.4%)	3183(53.6%)	<0.0001
	No	481(53.0%)	426(47.0%)	
Heard about family planning	Yes	1666(44.2%)	2100(55.8%)	<0.0001
on the radio in the last few months	No	1571(51.0%)	1509(49.0%)	
Heard about family planning	Yes	150(36.1%)	265(63.9%)	<0.0001
from newspapers/magazines in the last 12 months	No	3086(48.0%)	3344(52.0%)	
Heard about family planning on TV in the last few months	Yes	195(39.9%)	294(60.1%)	0.001
	No	3042(47.9%)	3314(52.1%)	
Woman’s religious affiliation	Catholic	1129(43.1%)	1489(56.9%)	<0.0001
	Protestant	1637(52.5%)	1482(47.5%)	
	Seventh Day Adventist	361(41.1%)	517(58.9%)	
	Other	103(46.6%)	118(53.4%)	
Visited by family planning worker in the last 12 months	Yes	813(40.7%)	1184(59.3%)	<0.0001
	No	2424(50.0%)	2426(50.0%)	
Visited health facility in the last 12 months	Yes	2379(48.4%)	2539(51.6%)	0.004
	No	858(44.5%)	1071(55.5%)	
At health facility, told about family planning	Yes	1081(47.5%)	1194(52.5%)	0.265
	No	1298(49.1%)	1345(50.9%)	
Household wealth index	Poorest	667(51.7%)	622(48.3%)	<0.0001
	Poorer	730(50.4%)	719(49.6%)	
	Middle	654(45.8%)	774(54.2%)	
	Rich	599(44.1%)	760(55.9%)	
	richest	586(44.4%)	735(55.6%)	
Place of residence	Urban	516(44.3%)	648(55.7%)	0.028
	Rural	2720(47.9%)	2962(52.7%)	
Number of living children	0	349(98.3%)	6(1.7%)	<0.0001
	1	607(49.6%)	618(50.4%)	
	2	498(42.7%)	867(57.3%)	
	3	1137(4.3%)	1427(55.7%)	
Husband/partner desires children	Same number	430(51.9%)	398(48.1%)	<0.0001
	More	1886(45.5%)	2261(54.5%)	
	Fewer	563(46.0%)	660(54.0%)	
	Do not know	358(55.2%)	290(44.8%)	
Husband/partner education	No education	601(51.9%)	558(48.1%)	0.018
	Primary	2222(46.3%)	2579(53.7%)	
	Secondary	295(47.0%)	333(53.0)	
	Tertiary	111(45.2%)	132(54.2%)	
	Don’t know	6(50.0)	6(50.0%)	

**Table 4:** Model comparison based on Deviance Information Criteria (DIC).

Statistics	Model 1	Model 2	Model 3	Model 4
DIC	8488.64	8560.34	8465.45	8462.39
$\bar{D}$	8428	8486.14	8388	8362.12
pD	29.92	37.10	38.73	50.133

**Table 5:** Summary of fixed effects of factors associated with women of childbearing age.

Variable	Posterior Estimate of the Mean	Posterior Standard Error	Odds Ratio	95% Credible Interval (CI)
**Intercept**	−5.3585	0.4743	0.0047	(0.0017 0.0112)
**Woman’s education (No education = reference)**			1.0000	
**Tertiary**	0.5841	0.1909	1.7934	(1.2355 2.61300)
**Secondary**	0.3157	0.1193	1.3371	(1.0853 1.7331)
**Primary**	0.1600	0.0752	1.1735	1.0125 1.3599)
**Wealth quintile (Poorest = reference)**			1.0000	
**Richest**	0.3598	0.1023	1.4330	(1.1727 1.7517)
**Richer**	0.2998	0.0887	1.3496	(1.1340 1.6063)
**Middle**	0.2201	0.0857	1.2462	(1.0533 1.4745)
**Poorer**	0.0703	0.0834	1.0728	(0.9107 1.2636)
**Visited health facility in the last 12 months (No = reference)**				
**Yes**	0.1960	0.0598	1.2165	(1.0820 1.3681)
**Currently residing with husband (Living = reference)**				
**Stays elsewhere**	−0.5811	0.0882	0.5593	(0.4702 0.6647)
**Number of living children (4 and more = reference)**				
**3**	−0.1217	0.0837	0.8854	(0.7512 1.0435)
**2**	−0.3055	0.0935	0.7368	(0.6131 0.8848)
**1**	−0.8057	0.1176	0.4468	(0.3544 0.5625)
**0**	−4.9371	0.4299	0.0072	(0.0029 0.0156)
**Religion (Others = reference)**				
**Catholic**	−03459	0.0585	0.7047	(0.6308 0.7936)
**Protestant**	0.1338	0.0854	1.1432	(0.967 1.3523)
**Seventh Day Adventist**	0.0211	0.1513	1.0213	(0.7600 1.3761)
**Respondent currently working (No = reference)**				
**Yes**	0.2394	0.0795	1.2705	(1.0868 1.4850)
**Heard about family planning on the radio in the last few months (No = reference)**				
**Yes**	0.1109	0.0554	1.1173	(1.0021 1.2455)
**Visited by family planning worker in the last 12 months (Yes = reference)**				
**No**	0.1960	0.0598	1.21653	(1.0812 1.3681)
**Heard about family planning from newspapers/magazines (No = reference)**				
**Yes**	0.2884	0.1197	1.3343	(1.0564 1.6896)
**Province (West = reference)**				
**Kigali**	0.0548	0.1047	1.0563	(0.4702 1.5038)
**South**	−0.1224	0.1066	0.8848	(0.8601 1.2969)
**North**	0.3580	0.1141	1.4305	(1.1436 1.7898)
**East**	0.1704	0.1051	1.1858	(0.9649 1.4579)
**Husband desires children (Same number = reference)**				
**More**	0.2622	0.0818	1.2998	(1.1069 1.5258)
**Fewer**	−0.0456	0.1153	0.9554	(0.7619 1.1979)
**Do not know**	0.2073	0.0964	1.2304	(1.0182 1.4866)

**Table 6:** Districts of Rwanda and their codes used in RDHS 2014/15.

Code	District	Code	District	Code	District	Code	District
1	Nyarugenge	9	Ruhango	17	Rusizi	24	Rwamagana
2	Gasabo	10	Muhanga	18	Nyamasheke	25	Nyagatare
3	Kicukiro	11	Kamonyi	19	Rulindo	26	Gatsibo
4	Nyanza	12	Karongi	20	Gakenke	27	Kayonza
5	Gisagara	13	Rubavu	21	Musanze	28	Kirehe
6	Nyaruguru	14	Rubavu	22	Burera	29	Ngoma
7	Huye	15	Nyabihu	23	Gicumbi	30	Bugesera
8	Nyamagabe	16	Ngororero				
